# Anakinra for tocilizumab-refractory febrile infection-related epilepsy syndrome with normal IL-1β levels: a case report

**DOI:** 10.3389/fimmu.2026.1866775

**Published:** 2026-06-12

**Authors:** Lijun Zhang, Deyuan Li, Jianjun Wang, Xiao Wang, Wanling Zhao, Guoyan Lu

**Affiliations:** 1 Department of Pediatrics, Ministry of Education Key Laboratory of Women and Children’s Diseases and Birth Defects, West China Second University Hospital, Sichuan University, Chengdu, Sichuan, China; 2 Department of Pediatrics, West China Second University Hospital (WCSUH)-Tianfu·Sichuan Provincial Children’s Hospital, Meishan, Sichuan, China

**Keywords:** anakinra, child, FIRES, IL-1β, IL-6, status epilepticus, tocilizumab

## Abstract

**Background:**

Febrile infection-related epilepsy syndrome (FIRES) is a severe epileptic encephalopathy with limited treatment options. Neuroinflammation, particularly involving IL-1β and IL-6, is a key driver. Evidence on sequential use of IL-6 receptor blockade (tocilizumab) followed by IL-1 receptor blockade (anakinra) is scarce, especially when IL-1β levels are normal.

**Case presentation:**

A previously healthy 6-year-old boy developed super-refractory status epilepticus six days after influenza A infection. He failed multiple antiseizure medications, anesthetics, corticosteroids, intravenous immunoglobulin, ketogenic diet, and mild therapeutic hypothermia. Cerebrospinal fluid (CSF) and serum cytokine testing showed elevated IL-6 but normal IL-1β. After initial improvement with tocilizumab, seizures relapsed. Tocilizumab retreatment and plasma exchange were ineffective. Switching to daily subcutaneous anakinra led to rapid seizure control within five days. He was weaned off anesthetics, extubated, and eventually discharged with mild cognitive dysfunction (PCPC score 2). At one-month follow-up, he had rare brief seizures and attended school.

**Conclusion:**

This case demonstrates that anakinra can be effective in tocilizumab-refractory FIRES even when CSF and serum IL-1β levels are normal. Anakinra should be considered as a second-line option in selected patients, regardless of IL-1β biomarker status.

## Introduction

Febrile infection-related epilepsy syndrome (FIRES) refers to the onset of seizures within 24 hours to 2 weeks after a prodromal fever, which rapidly progresses to super-refractory status epilepticus, presenting as new-onset refractory status epilepticus. It most commonly affects previously healthy children aged 2–17 years (1–3). The clinical manifestations are typically focal seizures that may secondarily generalize to bilateral tonic-clonic seizures. During the acute phase, seizures are difficult to control despite the use of multiple antiseizure medications, often requiring admission to the intensive care unit (ICU), and even intravenous administration of general anesthetics and invasive mechanical ventilation (3). The diagnosis requires the exclusion of other etiologies such as autoimmune encephalitis, intracranial infection, and genetic or metabolic encephalopathies. Early brain imaging may show no abnormalities, but abnormal signals in the basal ganglia, thalamus, hippocampus, and cerebral cortex can also be observed (4). Electroencephalography (EEG) features include diffuse slow waves, with multifocal seizures during the ictal phase. The prognosis is generally poor, with a mortality rate as high as 12%, and few patients return to their premorbid state (5). Therefore, early recognition and development of effective individualized treatment strategies are critical.

Given that the pathogenesis of FIRES remains unclear, standardized diagnostic and treatment protocols are still lacking. Neuroinflammation caused by dysregulation of the innate immune system is one of the leading hypotheses (3, 6). Therefore, in addition to combination antiseizure medications and/or anesthetics, first-line immunomodulatory therapies such as corticosteroids, high-dose intravenous immunoglobulin, and plasma exchange are often recommended early in the course (3, 7). However, their clinical efficacy is often unsatisfactory.

Consequently, second-line targeted therapies against neuroinflammation in FIRES patients have emerged. Preclinical and clinical studies have confirmed the association between neuroinflammation and epilepsy. Multiple cytokines are believed to be associated with the pathogenesis of epilepsy, particularly IL-1β, a member of the interleukin-1 gene family. IL-1β binds to type 1 IL-1 receptor (IL-1R1), activating downstream NF - κ B and MAPK signaling cascades, leading to the production of pro-inflammatory mediators. IL-1 receptor antagonist (IL-1RA) is an endogenous competitive antagonist of IL-1R1, which can block IL-1β activity (8, 9). Numerous studies have shown that IL-1RA can exert potent anticonvulsant and seizure suppression effects through selective inhibition (10, 11). Anakinra, as a human recombinant IL-1 receptor antagonist, plays an important role in autoimmune and inflammatory diseases (12). Several reports have indicated significantly elevated levels of inflammatory cytokines (IL-6, IL-1β, etc.) in the cerebrospinal fluid (CSF) and serum of FIRES patients, with CSF levels often higher than contemporaneous serum levels (13–15). The ketogenic diet, which can inhibit inflammatory pathways, prevent mitochondrial and neuronal injury, and improve seizure outcomes, is also recommended for early use in pediatric epilepsy patients including FIRES (16, 17). Notably, the targeted use of the IL-6 receptor antagonist tocilizumab and the IL-1β receptor antagonist anakinra in FIRES patients has gained increasing attention (8, 18–20). Although monotherapy or sequential use of tocilizumab and/or anakinra has achieved some efficacy, definitive conclusions remain lacking. We report a case of a child with FIRES who initially responded to tocilizumab in the acute phase but experienced a relapse, and whose seizures were subsequently controlled after switching to anakinra. Interestingly, cytokine testing in this child’s CSF and serum revealed only elevated IL-6, with normal IL-1β levels. The patient had a favorable prognosis at one−month follow−up after discharge.

## Case presentation

A previously healthy 6−year−old boy was admitted due to new−onset seizures 6 days after developing fever and being diagnosed with influenza A. The seizures manifested as tonic−clonic convulsions of all four limbs, accompanied by unresponsiveness, staring eyes, trismus, cyanosis of the lips, and foaming at the mouth. Each seizure lasted from several tens of seconds to up to 3 minutes, with a confused state between seizures. He rapidly progressed to refractory status epilepticus and was admitted to the ICU, where invasive mechanical ventilation was initiated on hospital day 2.

Initial treatment included phenobarbital, levetiracetam, midazolam, methylprednisolone (1 mg/kg/day for 3 days), intravenous immunoglobulin (2 g/kg), a ketogenic diet (initiated on day 5; using the classic 4:1 ratio of fat to carbohydrates plus protein, maintaining blood ketones at 2.0-5.0 mmol/L during the acute phase), and mild therapeutic hypothermia (33-34 °C for 27 days, with coagulation function and immune function dynamically monitored, all results remaining within acceptable ranges) (**Supplementary Document 1**). Because seizures continued to recur, intravenous sodium valproate and propofol were added (**Figure 1**). When the ketogenic diet was initiated on hospital day 5, the propofol infusion was gradually tapered and discontinued to avoid prolonged co−administration. During concurrent ketogenic diet and propofol administration, the clinical team closely monitored for signs of propofol infusion syndrome (PRIS). This monitoring included daily serum lactate and blood gas analysis, as well as regular assessment of serum lipids, creatine kinase, and cardiac function (21). Throughout the treatment period, no clinical or laboratory evidence suggestive of PRIS was observed (**Supplementary Document 1**).

**Figure 1:**
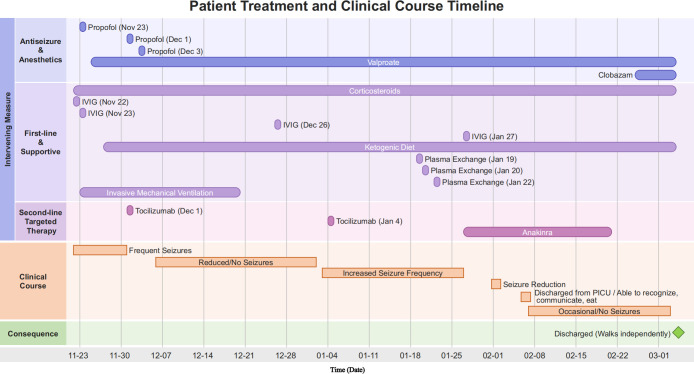
Treatment course of patient throughout hospitalization.

Routine laboratory tests, including complete blood count, C−reactive protein, procalcitonin, blood biochemistry, blood gas analysis, and metabolic screening (blood and urine), showed no significant abnormalities (**Table 1**). Cerebrospinal fluid (CSF) analysis revealed: white blood cells 3 × 10^6^/L, protein 332 mg/L, glucose 3.47 mmol/L (simultaneous blood glucose 4.5 mmol/L). CSF culture and metagenomic next−generation sequencing (mNGS) were negative for bacteria, viruses, fungi, and parasites. Autoimmune encephalitis antibody panels (anti−NMDAR, anti−LGI1, anti−CASPR2, anti−GAD65, anti−AMPAR, anti−Hu, anti−GABA_a_R, and anti−GABAB_r_) in both blood and CSF were negative. Brain magnetic resonance imaging (MRI) performed after admission showed mild widening of the bilateral ventricular bodies, abnormal signals in the right cerebral cortex and around the anterior and posterior horns of the lateral ventricles, and reduced bilateral hippocampal volume with increased T2−FLAIR signal (**Figures 2A, B**). Electroencephalography (EEG) revealed: continuous low−amplitude fast activity over the left fronto−central, left centro−parietal, and left parieto−occipito−posterior temporal regions, with gradually increasing amplitude; this activity slowly spread to adjacent left−sided leads and then evolved into low−to−moderate amplitude fast rhythm over the entire left hemisphere. Moderate−amplitude theta rhythm was seen over the left hemisphere, intermixed with moderate−amplitude sharp and spike waves, with slowing of the frequency and spread to the right leads. Concurrently, electromyographic (EMG) bursts were recorded from both deltoid muscles. During the waking or sleep transition period, the diffuse medium-high amplitude 1.5–4 Hz δ-θ slow waves were widely distributed, especially in the prefrontal and frontal regions. During sleep, repetitive sharp waves and spike waves were seen in the left or right forehead, forehead, central and temporal regions. At the same time, a small amount of low amplitude fast wave rhythm can be seen in the frontal-central, central-top and midline regions (**Figures 3A, B**).

**Table 1:** The main laboratory results of the patient on admission.

Items	Value	Reference range	Items	Value	Reference range
Blood routine			Cerebrospinal fluid		
WBC counts (10^9/L)	6.8	4.4-11.9	Cells (10^6/L)	3	<15
Neutrophil (%)	58.4	22-65%	Protein (mg/L)	332	80-430
Hemoglobin (g/L)	119	112-149	Glucose (mmol/L)	3.47	2.5-4.5
Platelet (10^9/L)	150	128-420	Chloride (mmol/L)	120	120-130
C-reactive protein(mg/L)	<0.2	0-8	CSF culture	Negative	Negative
Procalcitonin	0.02	0-0.5ng/ml	Metabolic disease		
ESR (mm/H)	4.5	<21	Blood	Negative	Negative
D-dimer (mg/L)	0.57	<0.55	Urine	Negative	Negative
CSF-cytokine			Blood-cytokine		
IL-Iβ (pg/ml)	<2.5	<12.3	IL-Iβ (pg/ml)	2.04	<12.3
IL-2 (pg/ml)	<2.5	<11.5	IL-2 (pg/ml)	<1.5	<11.5
IL-6 (pg/ml)	13.64	<12	IL-6 (pg/ml)	168.73	<20
IL-8 (pg/ml)	29.97	<22.8	IL-8 (pg/ml)	16.49	<21.4
IL-10 (pg/ml)	<2.5	<6.4	IL-10 (pg/ml)	<1.5	<5.9
IL-17 (pg/ml)	<10.0	<20.9	IL-17 (pg/ml)	<1.5	<20.6
IFN-α (pg/ml)	<2.5	<7.8	IFN-α (pg/ml)	<1.5	<7.9
IFN-γ (pg/ml)	<2.5	<17.5	IFN-γ (pg/ml)	<1.5	<17.3

WBC, white blood cell; ESR, erythrocyte sedimentation rate; PaO2, arterial partial pressure of oxygen; CSF, cerebrospinal fluid; IL-1β, Interleukin-1β.

**Figure 2:**
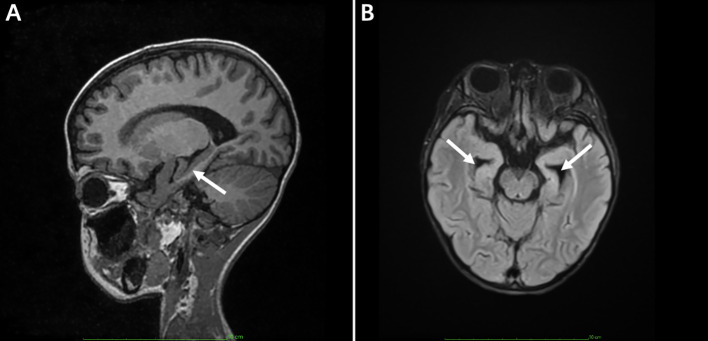
Imaging manifestations. **(A)** The hippocampal volume is reduced in cranial magnetic resonance imaging (MRI). **(B)** T2 weighted imaging (T2WI) shows high signal intensity.

**Figure 3:**
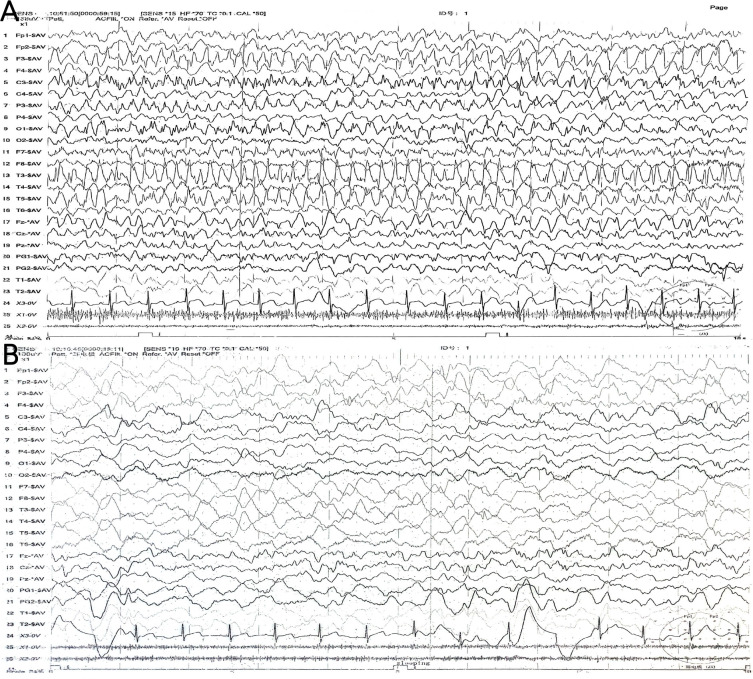
EEG findings. **(A, B)** Continuous low−amplitude fast waves were observed over the left fronto−central, left centro−parietal, and left parieto−occipito−posterior temporal regions, with gradually increasing amplitude. This activity slowly spread to adjacent left−sided leads and then evolved into a low−to−moderate amplitude fast rhythm over all left−sided leads. Moderate−amplitude theta rhythm was seen over the entire left hemisphere, intermixed with moderate−amplitude sharp and spike waves, with slowing of the frequency and spread to the right leads. Simultaneously, electromyographic (EMG) bursts were recorded from both deltoid muscles. **(B)** During sleep, repetitive sharp waves and spike waves were seen in the left or right forehead, forehead, central and temporal regions. At the same time, a small amount of low amplitude fast wave rhythm can be seen in the frontal-central, central-top and midline regions.

The child was diagnosed with FIRES. Because first−line immunomodulatory and antiseizure therapies were ineffective, cytokine testing was performed on blood and CSF on day 7 of admission. CSF analysis showed elevated IL−6 (13.64 pg/mL; reference <12) and elevated IL−8 (29.97 pg/mL; reference <22.8), while IL−1β was normal (<2.5 pg/mL; reference <12.3). Blood cytokine testing revealed elevated IL−6 (168.73 pg/mL; reference <20), whereas IL−1β was normal (2.04 pg/mL; reference <12.3).

On hospital day 9, tocilizumab (8 mg/kg intravenously) was administered. Seizures were gradually decreased and were controlled by hospital day 14, allowing gradual weaning of anesthetics.The patient was successfully extubated on hospital day 29. However, on hospital day 43, seizures recurred frequently of 5–10 per day. On hospital day 44, a second dose of tocilizumab (8 mg/kg) was given, and three sessions of plasma exchange were performed over the following week, but there was no sustained improvement.

On hospital day 60, treatment was switched to anakinra (2 mg/kg/day subcutaneously). By hospital day 65, seizures were fully controlled, and the patient’s level of consciousness markedly improved: he could recognize family members, follow simple commands, and eat orally. On hospital day 72, he was transferred to the neurorehabilitation department for continued care. Anakinra was discontinued on hospital day 91 (total duration 31 days) (**Figure 1**). While taking four oral antiseizure medications (levetiracetam, valproate, clobazam, and phenobarbital), he had no further frequent seizures. He was discharged on hospital day 102, at which time he could walk independently and perform simple activities of daily living.

At the nearly three months follow−up after discharge, while taking four antiseizure medications daily, he experienced approximately two brief seizure per month, each lasting a few seconds, with clear consciousness afterward. The patient started attending school of appropriate age about four months after the onset of acute FIRES, but experienced mild cognitive impairment, manifested as near memory loss. His Pediatric Cerebral Performance Category (PCPC) score was 2, indicating mild disability.

## Discussion

We present a child with tocilizumab-refractory FIRES in whom anakinra rapidly terminated status epilepticus and enabled return to near-premorbid function. This case has two key novel aspects (1): efficacy of anakinra after tocilizumab failure, and (2) clinical response despite normal CSF and serum IL-1β levels.

The first report of anakinra in FIRES was published in 2016 (13). Another retrospective study indicated that among 25 children with FIRES treated with anakinra, 11 had a >50% reduction in seizure frequency within one week of treatment, and early use significantly shortened the duration of mechanical ventilation, ICU stay, and total hospital stay (14). Similarly, our patient rapidly discontinued intravenous antiseizure medications and anesthetics after anakinra and was transferred to the general ward sooner.

Conversely, a 2020 study reported a 6−year−old boy diagnosed with FIRES who failed first−line antiseizure medications and also failed early anakinra but responded dramatically to tocilizumab, with elevated CSF IL−6 and IL−8 levels (15). These studies suggest the great therapeutic promise of targeted cytokine blockade in children with FIRES. However, there are few reports and no clear conclusions regarding whether anakinra and tocilizumab should be used alone or in combination, and the optimal sequence of their use. Our case provides support for anakinra as a rescue therapy after tocilizumab failure.

Notably, in our case, only CSF and serum IL-6 levels were elevated, while IL-1β levels were normal. In previous reports, anakinra-responsive FIRES cases were often associated with elevated CSF and/or serum IL-1β levels [8, 9]. Whether anakinra is recommended in children with normal IL-1 levels in CSF or serum has rarely been reported and remains inconclusive. Our patient had consistently normal IL-1β levels, yet anakinra was clearly beneficial. Regarding anakinra dosing, we administered anakinra subcutaneously at a dose of 2 mg/kg/day based on the patient’s age, weight, and clinical stability. The treating team selected this conservative initial dose without upward titration to minimize potential adverse effects, including infectious risk and cytopenias, while closely monitoring clinical response and laboratory parameters. As in a previous report (8), rapid seizure control was achieved with this low−dose regimen, and no dose escalation was required. Several explanations for the efficacy of anakinra despite normal IL−1β levels are possible (1): CSF IL-1β levels may not fully reflect compartmentalized IL-1 activity in the brain parenchyma (22) (2); anakinra might have off-target anti-inflammatory effects, e.g., on the IL-1 receptor downstream signaling independent of ligand concentration (9). To our knowledge, this is among the few reports of anakinra efficacy in FIRES with normal IL-1β, suggesting that biomarker-driven exclusion of anakinra may be premature. Our case suggests that anakinra may be a second-line option for children with FIRES even when CSF and/or serum IL-1β levels are normal.

Furthermore, considering the patient’s persistent super−refractory status epilepticus and the need to maintain neuroprotection while other therapies were being escalated, we extended the duration of mild therapeutic hypothermia. At approximately two weeks of treatment, we attempted to slowly wean therapeutic hypothermia, but seizure activity markedly worsened during the weaning process. We therefore decided to continue prolonged mild hypothermia. Prolonged mild hypothermia as a neuroprotective strategy has been reported in other conditions (23–25). During the extended period, we closely monitored for potential complications, including coagulopathy and immunosuppression. The patient did not experience any clinically significant adverse events; routine laboratory parameters (coagulation profile, blood gas analysis, immune function) remained within acceptable ranges (**Supplementary Document 1**). Further studies are needed to validate the feasibility and efficacy of prolonged mild hypothermia in FIRES. Regarding the dosage of corticosteroids, our dose of methylprednisolone (1 mg/kg/day for 3 days) is lower than the conventional pulse regimen (e.g. 20–30 mg/kg/day). This was decided after multidisciplinary discussions, including neurology specialist. Firstly, the initial diagnosis of the patient effectively excluded neurological diseases with strong evidence, such as necrotizing encephalopathy and autoimmune encephalitis; In addition, previous studies have shown that high−dose corticosteroid pulses therapy may not provide additional benefits to FIRES (26, 27). Secondly, the patient had a persistent fever upon admission, and high-dose steroids may have exacerbated the infection or promoted its spread. Thirdly, we are also concerned about ICU acquired weakness (ICU-AW), which may prolong the offline time for invasive mechanical ventilation. Therefore, we chose a lower dose to balance potential efficacy with the risk of infection and ICU-AW. No adverse events related to corticosteroids were observed.

Several limitations should be acknowledged. The lack of continuous EEG monitoring is one of the major limitations of our study. This may have limited our ability to detect subclinical or nonconvulsive status epilepticus during deep sedation. Although clinical observation and intermittent EEG assessments suggested that seizures were controlled, unrecognized subclinical activity may still have occurred. In addition, due to the patient’s financial constraints, we were unable to perform dynamic monitoring of cytokine levels in CSF and serum. Genetic testing for epilepsy or neuroinflammatory disorders was not conducted due to financial constraints. Third, because the patient received concurrent therapies (e.g., ketogenic diet, plasma exchange), a causal relationship between anakinra and seizure control cannot be definitively established; however, a strong temporal correlation exists between the initiation of anakinra and seizure control. Finally, the follow−up period was only three months, which is insufficient to assess long−term seizure freedom, cognitive outcomes, or durability of the treatment response. Longer observation is needed to determine the ultimate prognosis.

## Conclusion

This case provides new evidence that anakinra may be effective in tocilizumab-refractory FIRES, even when IL-1β levels are normal. Future prospective, multicenter studies are needed to define optimal sequential or combination strategies targeting IL-1 and IL-6 pathways in FIRES.

## Data Availability

The original contributions presented in the study are included in the article/**Supplementary Material**. Further inquiries can be directed to the corresponding authors.
