# Supplementary material for: Anakinra for tocilizumab-refractory febrile infection-related epilepsy syndrome with normal IL-1β levels: a case report
Source: Front Immunol. 2026 Jun 12;17:1866775. doi: 10.3389/fimmu.2026.1866775 (PMC13303200; doi:10.3389/fimmu.2026.1866775)
Supplement: Supplementary file 1 [file Table1.docx]

| Items | Value | Reference range | Items | Value | Reference range |
| --- | --- | --- | --- | --- | --- |
| Cardiac function | | | Creatine kinase | | |
| LVEF | 62% | 55% - 80% | CK (U/L) | 28.2-29.2 | 24-190 |
| FS | 31% | 28%-44% | CK-MB (ng/mL) | 0.76-1.23 | <6.36 |
| Arterial blood gas analysis | | | Blood lipid | | |
| PH | 7.36-7.42 | 7.35-7.45 | HDL (mmol/L) | 1.0-1.6 | >0.9 |
| HCO3- (mmol/L) | 19.4-24 | 22-27 | LDL (mmol/L) | 2.6-3.1 | 0-3.12 |
| BE (mmol/L) | (-6) - (2) | (-2) - (-3) | TG (mmol/L) | 0.5-1.8 | 0-2.3 |
| Lac (mmol/L) | 0.32-1.32 | 0.36-1.25 | CHO (mmol/L) | 3.9-5.6 | ≤5.6 |
| Coagulation function | | | Immunoglobulin | | |
| PT(s) | 10-12.6 | 9.1-15.1 | IgG (g/L) | 21.3 | 8.0-17 |
| INR | 0.92-1.18 | 0.8-1.5 | IgM (g/L) | 1.6 | 0.6-2.6 |
| aPTT (s) | 23.6-30.5 | 18.3-38.3 | IgA (g/L) | 2.19 | 0.72-4.29 |
| Fibrinogen (mg/dL) | 68-307 | 200-400 | IgE (IU/mL) | 26.4 | <90 |
| D-dimer (mg/L) | 0.48-0.81 | <0.55 | C3 (g/L) | 1.45 | 0.78-2.10 |
| FDP (ug/mL) | 2.5-5.6 | <5 | C4 (g/L) | 0.39 | 0.17-0.48 |
